# Inhibition shapes response selectivity in the inferior colliculus by gain modulation

**DOI:** 10.3389/fncir.2012.00067

**Published:** 2012-09-18

**Authors:** Joshua X. Gittelman, Le Wang, H. S. Colburn, George D. Pollak

**Affiliations:** ^1^Section of Neurobiology, Institute for Neuroscience, Center for Perceptual Systems, The University of TexasAustin, TX, USA; ^2^Department of Biomedical Engineering, Hearing Research Center, Boston UniversityBoston, MA, USA

**Keywords:** inhibition, spike threshold, modeling, response selectivity, gain control, directional selectivity

## Abstract

Pharmacological block of inhibition is often used to determine if inhibition contributes to spike selectivity, in which a preferred stimulus evokes more spikes than a null stimulus. When inhibitory block reduces spike selectivity, a common interpretation is that differences between the preferred- and null-evoked inhibitions created the selectivity from less-selective excitatory inputs. In models based on empirical properties of cells from the inferior colliculus (IC) of awake bats, we show that inhibitory differences are not required. Instead, inhibition can enhance spike selectivity by changing the gain, the ratio of output spikes to input current. Within the model, we made preferred stimuli that evoked more spikes than null stimuli using five distinct synaptic mechanisms. In two cases, synaptic selectivity (the differences between the preferred and null inputs) was entirely excitatory, and in two it was entirely inhibitory. In each case, blocking inhibition eliminated spike selectivity. Thus, observing spike rates following inhibitory block did not distinguish among the cases where synaptic selectivity was entirely excitatory or inhibitory. We then did the same modeling experiment using empirical synaptic conductances derived from responses to preferred and null sounds. In most cases, inhibition in the model enhanced spike selectivity mainly by gain modulation and firing rate reduction. Sometimes, inhibition reduced the null gain to zero, eliminating null-evoked spikes. In some cases, inhibition increased the preferred gain more than the null gain, enhancing the difference between the preferred- and null-evoked spikes. Finally, inhibition kept firing rates low. When selectivity is quantified by the selectivity index (SI, the ratio of the difference to the sum of the spikes evoked by the preferred and null stimuli), inhibitory block reduced the SI by increasing overall firing rates. These results are consistent with inhibition shaping spike selectivity by gain control.

## Introduction

To determine the role of inhibition in sensory processing, iontophoretic application of pharmacological agents during extracellular recording has been used for decades (Sillito, 1975; Moore and Caspary, 1983; Yang et al., 1992; Klug et al., 2002; Andoni et al., 2007; Kutscher and Covey, 2009). Typically, stimulus-evoked spikes are measured before and after application of the agent. Spike selectivity, a difference between the spikes evoked by a preferred stimulus compared to a less-preferred or null stimulus, is generally reduced following inhibition block.

A limitation is that extracellular electrodes only measure output spikes, and therefore the underlying mechanisms must be inferred. For example, blocking inhibition in the auditory midbrain nucleus, the inferior colliculus (IC), reduces spike selectivity for frequency, intensity, frequency modulations (FMs), duration, binaural sensitivity, and conspecific communication calls (Yang et al., 1992; Casseday et al., 1994; Fuzessery and Hall, 1996; Kautz and Wagner, 1998; Klug et al., 1999; Andoni et al., 2007). A common interpretation is that spike selectivity depends on differences between the preferred- and null-evoked inhibitions: differences in timing (temporal pattern), in size (total conductance), or both. These differences, combined with the differences between the preferred and null excitations, comprise what we term “synaptic selectivity.” Synaptic selectivity is required to create input selectivity, i.e., the differences between the total input current, the summation of all of the excitation and inhibition evoked by the preferred and null stimuli. Input selectivity then underlies spike selectivity.

Additionally, inhibition might enhance spike selectivity by modulating the neuronal gain i.e., the ratio of output (spikes) to input (current) (Ingham and McAlpine, 2005; Pérez-González et al., 2012). In cases where inhibition modulates spike selectivity by setting the gain, synaptic selectivity could rely on differences between the preferred and null excitations to generate input selectivity, and inhibition does not necessarily contribute to synaptic selectivity. Instead, inhibition reduces the input current, which can reduce output spikes and also modulate the gain. For example, inhibition could enhance spike selectivity by keeping the null inputs below spike threshold, such that they evoke no spikes (gain = 0), but preferred inputs evoke spikes (Rose and Blakemore, 1974; Wehr and Zador, 2003; Gittelman et al., 2009; Katzner et al., 2011). After inhibitory block, both the preferred and null inputs get bigger. If null signals become suprathreshold (gain > 0), spikes are evoked and spike selectivity is reduced.

Inhibition might also enhance spike selectivity by keeping firing rates well below maximum (saturation) and in a range with high gain, where small input selectivity translates into large spike selectivity (Gittelman et al., 2009; Gittelman and Li, 2011). If blocking inhibition increases the inputs enough to approach saturation, null firing will increase more than the preferred, reducing or even eliminating the difference between the preferred and null spike counts. In this scenario, inhibition enhances spike selectivity even when the preferred and null inhibitions are identical and the synaptic selectivity mechanism is entirely excitatory. We term these inhibitory mechanisms that do not contribute to synaptic selectivity as “gain control,” and we note that roles for inhibition in gain control and synaptic selectivity are not mutually exclusive.

We previously reported that inhibition enhances FM selectivity in IC cells by keeping null responses below threshold (Gittelman et al., 2009; Gittelman and Pollak, 2011). This finding was independent of whether inhibition contributed to synaptic selectivity and is consistent with gain control described above. However, the conclusions were based solely on studies in one IC cell type (sustained cells with high input resistance), with a limited range of synaptic selectivity mechanisms and passive neuronal modeling (no voltage-gated channels, no spikes). In the current study, modeling was used to test a broader range of scenarios. Spiking models were designed with input/output (I/O) functions that span the range of IC cells measured *in vivo*. Selective spiking was created with a broad range of synaptic selectivity mechanisms, including two in which inhibition made no contribution to synaptic selectivity, i.e., there were no differences between the preferred and null inhibitions. We then removed inhibition from the models to determine whether the mechanisms underlying synaptic selectivity could be distinguished based on the changes in spike selectivities before and after blocking inhibition. Finally, previously reported data were analyzed using these spiking models to determine whether inhibition acting as a gain control was consistent with the effects of blocking inhibition on FM selectivity in the IC.

## Materials and methods

### 

#### In vivo

Membrane properties and FM-evoked conductance analyses from the cells presented here were reported previously (Gittelman et al., 2009). Responses to current steps and to FM sweeps were recorded using patch electrodes in whole-cell current-clamp mode from the central nucleus of the IC in awake *Tadarida brasilensis mexicana*. Input/output (I/O) functions were the number of spikes evoked in the first 50 ms during prolonged (200 ms) current step injections, as functions of current level. The eight conductance sets were derived from five sustained cells in which we recorded spikes and postsynaptic potentials (PSPs) evoked by a pair of FMs, a preferred and null FM that differed only in direction. In two cells we used a single FM pair in each cell to derive one conductance set per cell. In three cells we used two FM pairs in each cell to derive two conductance sets per cell, for a total of eight conductance sets (16 conductance pairs).

### Cell modeling

**Figure 1:**
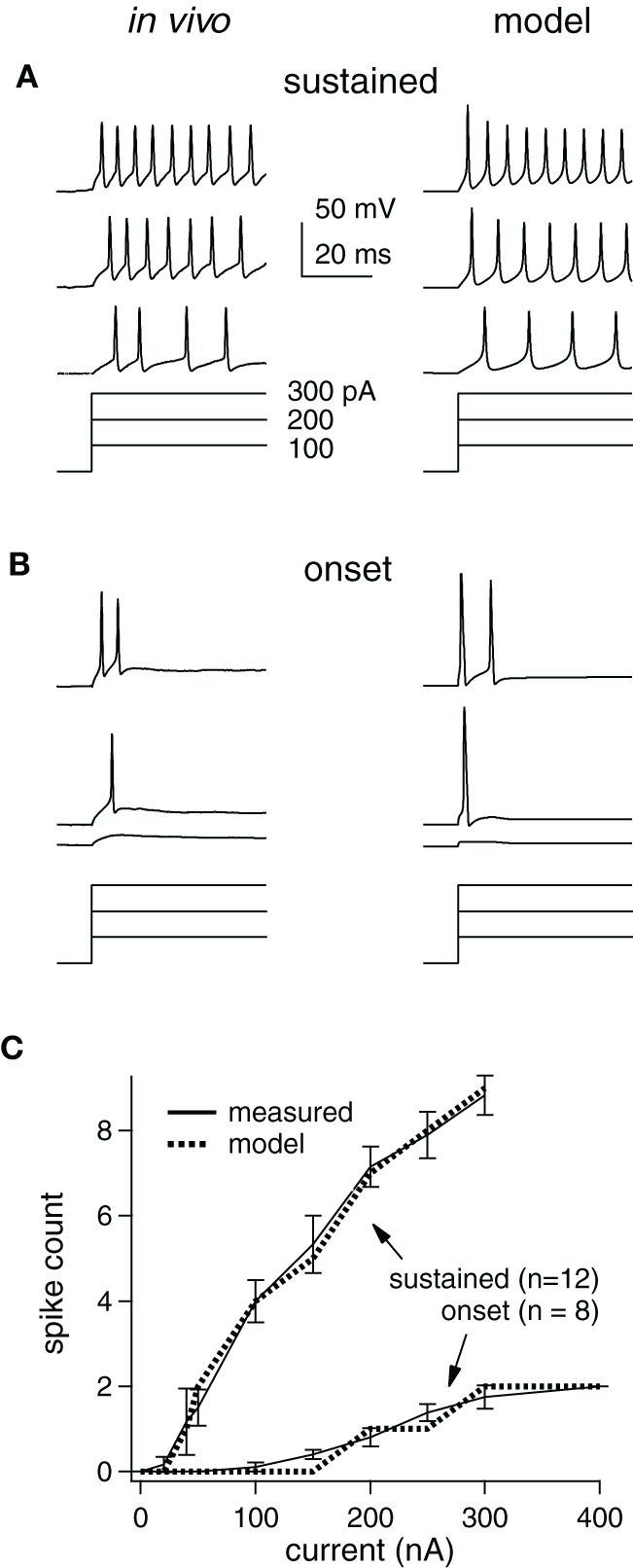
**Measured and modeled current-step responses match. (A)** *in vivo* (left) and model cell (right) potentials (top) for a sustained cell stimulated with current steps (bottom). Scale bars and step sizes also apply to **(B)**. **(B)** Same as **(A)** for an onset cell. **(C)** Empirical and model I/O functions for average response of each cell type.

**Figure 2:**
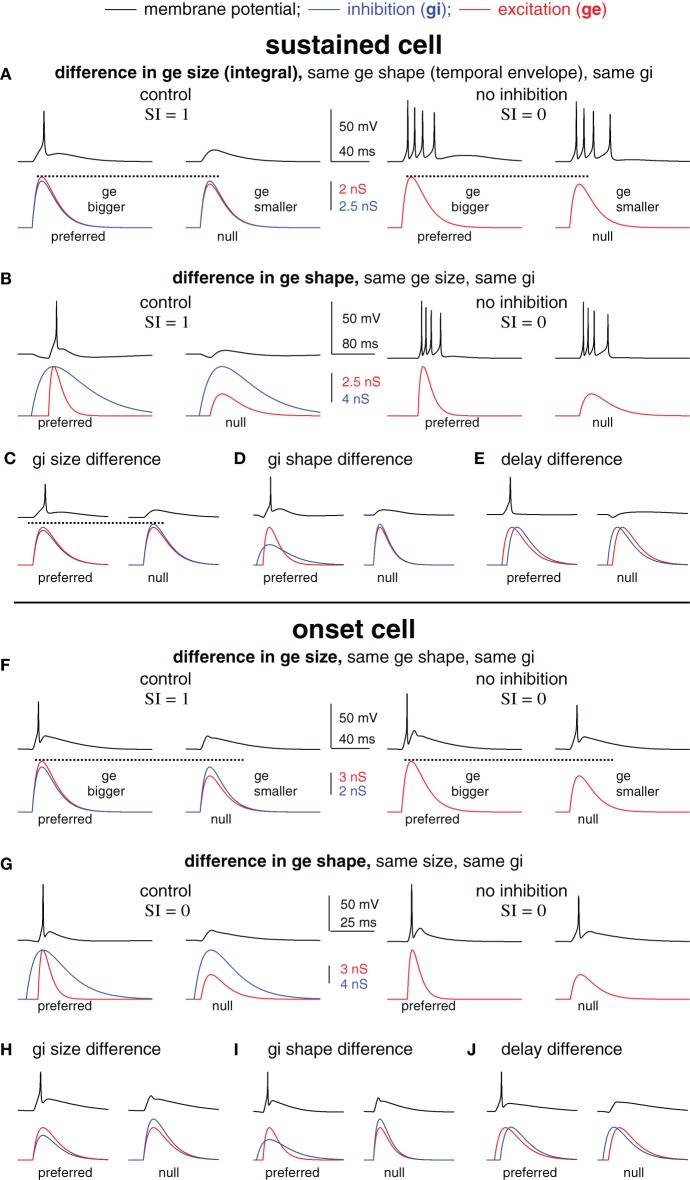
**Blocking inhibition can eliminate spike selectivity independent of the underlying mechanism of synaptic selectivity.** Spike selectivity is generated by five different mechanisms of synaptic selectivity in a modeled sustained cell **(A–E)** and onset cell **(F–J)**. Each mechanism had differences in only one synaptic conductance parameter, as shown. In each case, the preferred conductance pair evoked one spike and the null-evoked no spikes (selectivity index SI = 1). Removing inhibition eliminated spike selectivity (SI = 0) in every example.

**Figure 3:**
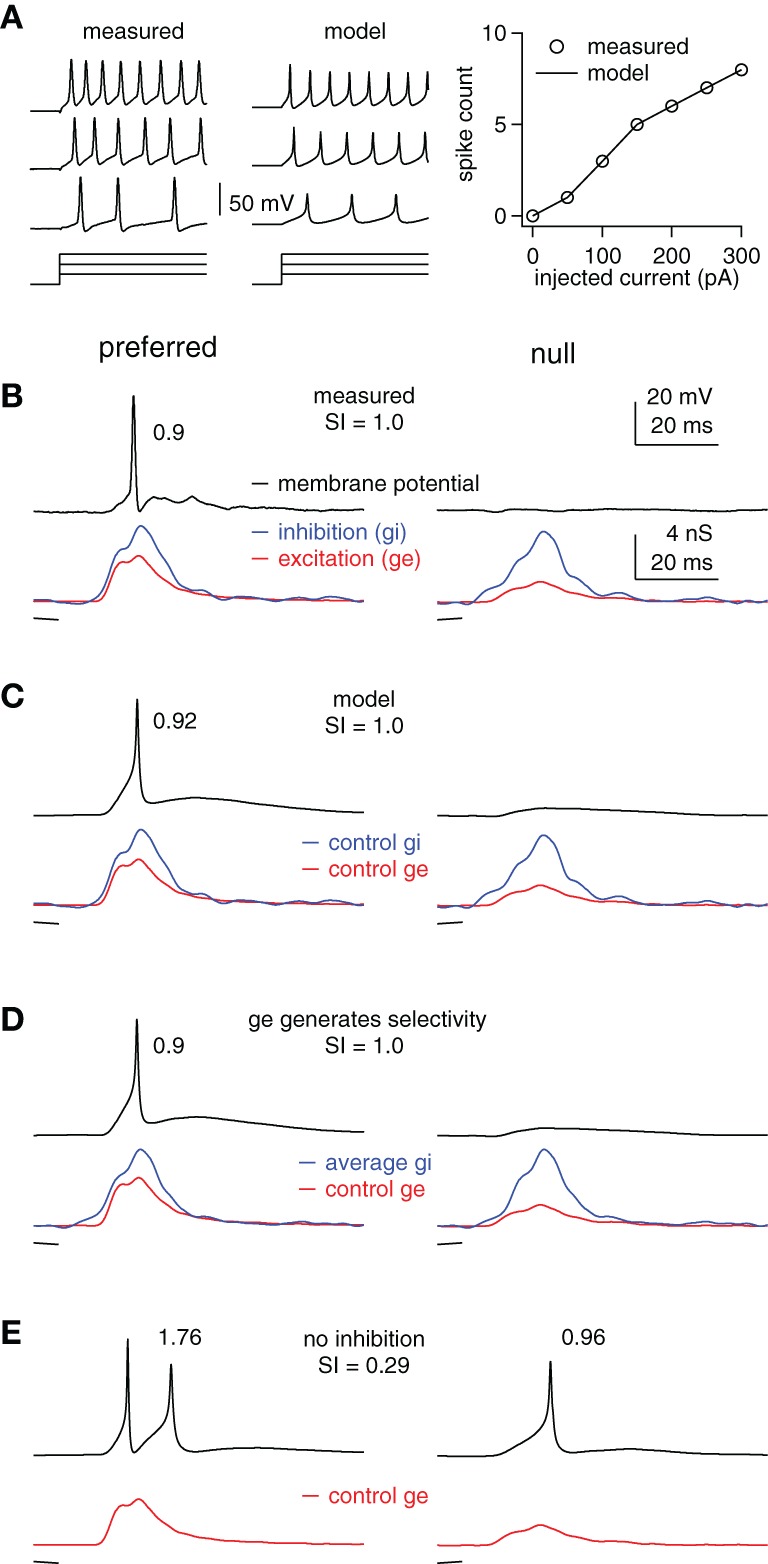
**Inhibition enhances FM selectivity by controlling the gain, example cell. (A)** Measured and computed responses to 100, 200, and 300 pA current steps (50 ms shown). Right: measured and computed I/O functions. **(B)** Measured membrane potentials (top) in response to preferred (downward, single trace) and null (upward, average of 10 traces) FMs and the derived FM-evoked synaptic conductance (bottom). Spike probability and SI as shown. **(C–E)** Using the model cell from **(A)** and the control excitations shown in **(B)**, we computed membrane responses using **(C)** the control inhibitions, **(D)** the average of the preferred and null inhibitions, and **(E)** no inhibition. Model firing probabilities and SIs as shown.

**Figure 4:**
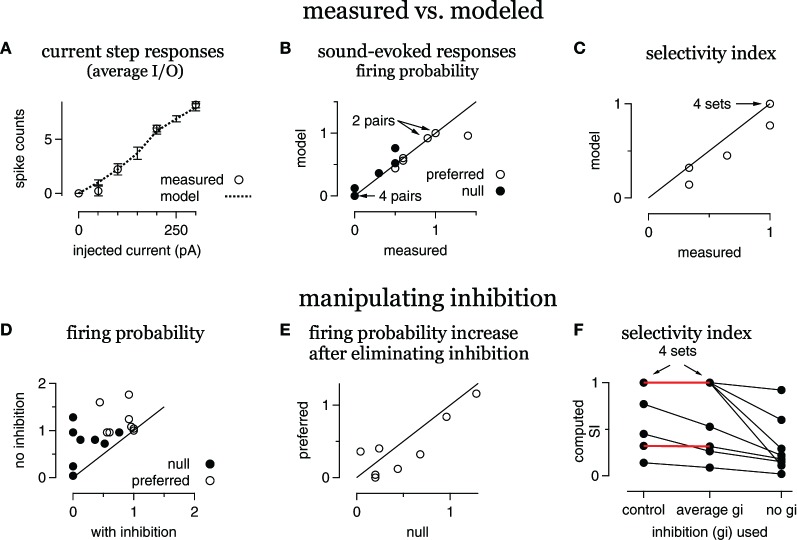
**Inhibition enhances FM selectivity by controlling gain, summary. (A)** Average modeled and measured I/O curves. **(B)** Measured FM-evoked spike probability plotted against spike probability computed in the models using the derived FM-evoked conductance pairs. **(C)** Selectivity index (SI) for all eight FM sets, modeled vs. measured. **(D)** Computed spike probability, with and without inhibition. **(E)** The change in computed spikes/trial following the elimination of inhibition. The change is equal to the spikes/trial under control conditions with inhibition intact subtracted from the spikes/trial with no inhibition. **(F)** Selectivity index computed using either the control conductance sets, conductance sets with the average inhibition and the control excitations, or with the control excitations only. Red lines indicate no change in SI using the average inhibition.

Single-compartment models comprised excitatory and inhibitory synaptic inputs, voltage-independent leak channels, and three voltage-dependent channels (Rothman and Manis, 2003): sodium (Na); a high-threshold potassium (KHT); and low-threshold potassium (KLT). Parameters were optimized to match empirically measured spiking, either average (Figures 1, 2), or individual neurons (Figures 3, 4). We assumed two synaptic conductances, one for excitation and one for inhibition with reversal potentials of 0 and −70 mV, respectively.

#### Average responses

We matched the average measured I/O functions of sustained and onset-burst cell types (Tan et al., 2007; Xie et al., 2008). The published voltage-dependent channels (Rothman and Manis, 2003) recreated the sustained responses. However, while the published KLT currents create onset responses, such as those recorded *in vitro* (Koch and Grothe, 2003), they do not generate the bursting pattern observed in the IC *in vivo* (Tan et al., 2007; Xie et al., 2008). Onset-burst cells can fire a burst of spikes at the start of a prolonged depolarization, rather than the single spike fired by onset cells recorded *in vitro*. In conjunction with KLT, a number of mechanisms could produce this burst pattern, such as hyperpolarization-activated currents (Ih) and *t*-type calcium currents (Kopp-Scheinpflug et al., 2011). However, our goal here was to match the I/O function in the simplest way possible, by extending the temporal window for spike initiation to enable bursting. To achieve the burst pattern, we changed KLT activation to a sigmoid-shaped temporal window W(t). Thus,

$$\bar{{\text{G}}_{\text{KLT}}}\left(\text{t}\right)=\bar{{\text{G}}_{\text{KLT}}}*\text{W}\left(\text{t}\right)=\frac{\bar{{\text{G}}_{\text{KLT}}}}{{\left(\text{1 + exp}(-(\text{t}-{\text{T}}_{\text{delay}}))\right)}^{\text{4}}},$$

where $\bar{{\text{G}}_{\text{KLT}}}$ is the maximum conductance for KLT, T_delay_ is the relative delay, and *t* is time (ms). This equation simulated the effect of delayed KLT activation because $\bar{{\text{G}}_{\text{KLT}}}(t)$ is close to 0 for *t* < T_delay_ and $\bar{{\text{G}}_{\text{KLT}}(\text{t})}$ rapidly increases to $\bar{{\text{G}}_{\text{KLT}}}$ for *t* > T_delay_. The timing and shape of W(t) determined the number of spikes in the burst before KLT activated sufficiently to curtail firing. We found a good match to the empirical data with T_delay_ = 5 ms. While perhaps not biophysically realistic, implementing W(t) achieved the goal of matching the measured onset-burst function. In our view, the results in this study are not affected by how the I/O function was achieved.

#### Individual cells

The average sustained model was adjusted to match empirical measurements from the individual sustained-type cells (Figures 3, 4). First, the model membrane capacitance and leak conductance were adjusted to match measured values. Then, the maximum voltage-gated conductances (G_Na_, G_KHT_, and G_KLT_) were systematically varied until the model spike counts matched the empirical spiking responses to FM sweeps and current steps. To simulate measured spike variability, we scaled the derived synaptic conductances by factors chosen randomly from a Gaussian distribution (mean = 1; SD = 0.05). Scaling the conductances would represent variation in the size of the postsynaptic currents, possibly due, for example, to variation in the afferent firing or in vesicle release. Models were then stimulated with 25 independent pairs of scaled synaptic conductances. Model spike probability, the average spikes/trial, was calculated and compared to measured spike probability.

#### Conductance waveforms

In Figure 2, both the excitatory and inhibitory conductance waveforms [g_E_(t) and g_I_(t) respectively] were simulated as the difference of two exponentials, i.e., ${\text{g}}_{\text{E}}\left(\text{t}\right)=\frac{{\text{G}}_{\text{E}}}{{\text{g}}_{\text{norm}}}\left[\exp\left(-\text{t/}\tau_{\text{E}}\right)-\text{exp}(-\text{t/}\tau_{\text{E}\_\text{rise}})\right]$, where G_E_ is the peak conductance, *t* is time (ms), τ_E_ and τ_E_rise_ are the decay and rising time constants (ms), respectively, with τ_E_>τ_E_rise_. The constant g_norm_ was chosen so that the maximum value of the conductance was G_E_. Specifically, the peak value of g_E_(t) occurs at time t_p_, where ${\text{t}}_{\text{p}}\text{=}\frac{\tau_{\text{E}\_\text{rise}}*\;\tau_{\text{E}}}{\tau_{\text{E}}-\tau_{\text{E}\_\text{rise}}}\text{ln}\frac{\tau_{\text{E}}}{\tau_{\text{E}\_\text{rise}}}$ and g_norm_ is the value of the difference in the exponentials at time t_p_, g_norm_ = exp(−t_p_/τ_E_) − exp(−t_p_/τ_E_rise_). The conductance parameters that we varied to generate selective responses were relative peak times, the conductance sizes (the integrated conductance functions) and the shapes (the time constants). Each conductance parameter was varied separately, once for excitation and once for inhibition. The integrated conductance was held fixed by adjusting the maximum conductance.

## Results

### Model cells match intrinsic firing properties measured *In vivo*

How synaptic selectivity, the differences between the preferred- and null-evoked synaptic inputs, translates into spike selectivity depends on the excitability of the postsynaptic cells. We therefore optimized model cells to match the spike counts evoked by prolonged current steps measured *in vivo* from 20 IC cells that were representative of the larger data set in (Xie et al., 2008) (Figure 1). IC cells have been categorized into three types that account for ~95% of the population in our hands: sustained, adapting, and onset (Koch and Grothe, 2003; Xie et al., 2008). We concentrated on the spike counts in the first 50 ms, since we ultimately examined responses to brief (<50 ms) signals. During this period, adapting and sustained cell spike counts are similar, and thus, we only present sustained and onset models [~85% of the cells in Xie et al. (2008)].

Examples of measured and computed spike waveforms and stimulating currents are shown for the sustained and onset cell (Figures 1A,B, respectively). The spike counts match well in both cell types, although the waveforms are somewhat different, especially in the sustained cell. In particular, spike heights in the sustained model decline, likely due to Na channel inactivation, and the membrane potential in between spikes is rather different. We suspect this is due to the limited implementation of voltage-gated channels and cell geometry, but we do not think that is has a major impact on our findings (see “Discussion”).

Single compartment models were optimized to match the average responses of these two cell populations (“average models”), and are shown as input/output (I/O) functions (Figure 1C). Although, we used current steps as stimuli, the I/O curve shape would be similar if the input current were synaptic input strength. We did not inject enough current to reach saturation in the *in vivo* study, but as expected the slopes become less steep with increasing stimulus strength (Darian-Smith, 1960; Tateno et al., 2004). We used these average model cells to examine the roles of inhibition in generating spike selectivity.

### Changes in spike counts after inhibitory block do not distinguish among five synaptic mechanisms in the model cells

We next created spike selectivity with five different mechanisms of synaptic selectivity (Figure 2) using the average sustained (top, **A–E**) and onset (bottom, **F–J**) cell models from Figure 1. These examples were designed to illustrate that amongst very diverse mechanisms of synaptic selectivity, the role(s) of inhibition are not always revealed by changes in spike counts following inhibitory block. In each case, synaptic selectivity was created by only a single difference between the preferred- and null synaptic inputs, as described below. Of the five cases, the mechanism underlying synaptic selectivity was entirely excitatory in two, entirely inhibitory in two, and in one case both excitation and inhibition contributed. However, inhibition reduced the current strength in all five cases, which modulated the gain, i.e., the ratio of output spikes to input current. As will be shown, spike selectivity was dependent on the relative gains of the preferred and null inputs. Consequently, eliminating inhibition from the model eliminated spike selectivity in every case.

In the first example, the mechanism underlying synaptic selectivity was entirely excitatory (sustained cell, Figure 2A, left; onset cell, Figure 2F, left). The only difference between the preferred- and null synaptic conductances was that the integral (size) of the preferred excitatory conductance was larger than the null. The preferred and null excitations were identical in temporal envelope (shape), and the preferred and null inhibitions were identical in both size and shape. Finally, there was no delay between the excitation and inhibition in either the preferred or null conductance pair. In this (and each) example, the synaptic conductances were made such that the preferred conductance pair evoked spikes but the null pair did not.

**Figure 5:**
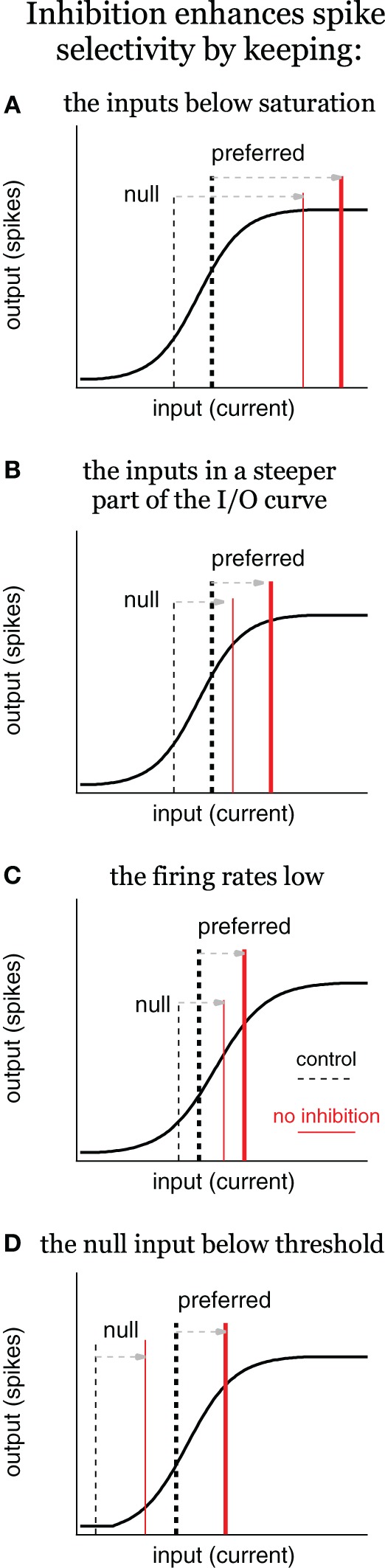
**Inhibition enhances spike selectivity by modulating gain, schematic.** Input/output (I/O) schematics illustrate three scenarios in which inhibition enhances spikes selectivity by reducing spike rates and controlling gain, the ratio of output/input. In each panel, the solid black line represents the I/O curve, the dashed vertical lines represent the input current for the control condition (tall and thick for preferred; short and thin for null), and the red lines represent the input current with no inhibition. Inhibition enhances spike selectivity by: **(A)** keeping the inputs below saturation; **(B)** keeping the inputs in the steepest part of the I/O curve; **(C)** keeping the firing rates low; and **(D)** keeping the null input below threshold, where the gain is zero. In **(C)**, it is not required for inhibition to change the gain to enhance spike selectivity, although, it does in this example.

We then show what would happen if inhibition were blocked. We simulated blocking inhibition by eliminating inhibition and stimulating with excitation only (Figures 2A,F right). In each model cell, the preferred and null excitation presented alone evoked identical spike counts. Indeed, they were designed to do so in order to illustrate the point that blocking inhibition can eliminate spike selectivity even when inhibition makes no contribution to synaptic selectivity. Consider that the preferred and null inhibitions were identical, and therefore, inhibition could not by itself create synaptic selectivity. Instead, the difference between the preferred and null excitations created synaptic selectivity. However, in the absence of inhibition, the synaptic selectivity did not translate into spike selectivity. In order to translate into spike selectivity, synaptic selectivity must generate a difference in the inputs (input selectivity) that falls in a region of the I/O curve that is sufficiently steep to generate a difference in the spike counts (see schematic in Figure 5). Here, we made the excitatory inputs so that when presented without inhibition the resulting inputs were both large, and thus, fell in the upper region of the I/O function that was not sufficiently steep to generate spike count differences. With inhibition, the inputs were reduced in amplitude, shifting their position leftward on the I/O function where the slope was steep enough to generate spike differences (in this case, the null generated no spikes). By reducing the strength of both the preferred and null inputs, inhibition modulated the gain. Spike selectivity was enhanced because the gain of the null was reduced to zero, whereas the preferred gain was still positive.

The second example was similar to the first except that synaptic selectivity was due to the difference in the temporal envelopes (shapes) of the preferred and null excitation, with no differences in the size (integral) of excitation, and no differences between the preferred and null inhibitions (sustained cell, Figure 2B; onset cell, Figure 2G). Again, the inputs were designed so that in the absence of inhibition the preferred and null excitations evoked identical spike counts (right). These (extreme) examples show that inhibition can enhance spike selectivity by modulating the gain without contributing to synaptic selectivity.

In the remaining three examples, synaptic selectivity depended on inhibition; there were no differences between the preferred and null excitations. Consequently, removing inhibition eliminated synaptic selectivity entirely, and therefore eliminated both input- and spike selectivity (not shown). In two cases the synaptic selectivity was entirely inhibitory; the preferred and null inhibitions differed either in size (total conductance) (sustained cell, Figure 2C; onset cell, Figure 2H) or shape (sustained cell, Figure 2D; onset cell, Figure 2I), and the preferred and null excitations were identical. The last example shows the classic case of creating spike selectivity by a difference in the delay between excitation and inhibition (sustained cell, Figure 2E; onset cell, Figure 2J).

We note that in addition to its contribution to synaptic selectivity, inhibition was also designed to enhance spike selectivity in each of the five cases as a gain control, i.e., by keeping the null inputs below threshold and by keeping the overall input strength well below saturation. We conclude from this section that in many scenarios the elimination of spike selectivity following inhibitory block does not reveal the mechanisms underlying synaptic selectivity. Importantly, the results were similar in the two different model cells that represent the extremes of the cell types found in the IC (Xie et al., 2008; Gittelman et al., 2009), suggesting that this may be true in many cell types.

### Blocking inhibition reduces spike selectivity for FM direction by adjusting the gain

We now examine how inhibition contributes to spike selectivity for FM direction in IC cells using the above modeling strategy with empirical data. We used this approach to assess the extent to which inhibition enhanced spike selectivity by contributing to synaptic selectivity as compared to acting as a gain control, and to determine whether the empirically derived model was consistent with the ubiquitous finding that blocking inhibition reduces spike selectivity and increases spike counts.

In this section we used previously published synaptic conductances that were derived from FMs presented in sets consisting of two identical sweeps, one that swept upward and one that swept downward. The “average” sustained model cell from Figure 1 was adjusted to match the empirically derived I/O functions for each individual cell from which FM-evoked conductances were derived. Each FM evoked a conductance pair consisting of an excitation and an inhibition. Thus, each FM set, consisting of a downward and an upward sweep, evoked two conductance pairs, one evoked by the preferred sweep direction, and another evoked by the null direction. We refer to the preferred- and null-evoked conductance pairs together as a conductance “set.” In three cells, two FM sets were presented, and in two cells only one FM set was presented. Thus, we derived two conductance sets each from three cells, and one conductance set each from two cells for a total of eight conductance sets, or 16 conductance pairs.

We show an example cell in Figure 3, followed by summary results in Figure 4. The computed and measured I/O functions of the example cell were identical (Figure 3A), and the FM-evoked spiking matched the spiking computed by stimulating the model with the FM-evoked conductance pairs (Figures 3B,C). The preferred FM reliably evoked spikes, whereas the null FM never evoked spikes (average spikes/trial of 0.9 and 0, respectively, Figure 3B). We calculated spike selectivity using the SI [SI = (P − N)/(P + N)], where P is the preferred spike count and N is the null spike count. The SI ranges from 0 (non-selective) to 1 (maximum selectivity). Here, SI = 1. Similar to the measured data, computed firing probability to the preferred conductance pair was 0.92, and computed firing to the null was 0 (SI = 1, Figure 3C).

Inspection of the FM-evoked synaptic conductance waveforms shows that the preferred excitation was larger than the null excitation, and they differed in shape. Additionally, the preferred inhibition was also slightly larger than the null inhibition, and they differed somewhat in shape (Figures 3B,C, bottom). We point this out to make it clear that both excitation and inhibition contributed to synaptic selectivity, but that it is not clear how inhibition's contribution to synaptic selectivity affected spike selectivity.

To determine the extent to which inhibition's contribution to synaptic selectivity affected spike selectivity, we eliminated the differences between the preferred and null inhibitions. We did this by averaging the preferred and null inhibitory conductance (Figure 3D). We then stimulated the model cell with a conductance pair comprised of the average inhibition and either the preferred or the null excitation. Under these conditions, inhibition makes no contribution to synaptic selectivity, but continues to modulate the input strength in approximately the same way as in the control condition.

In this model, the differences in excitation combined with the average inhibition generated the same firing probabilities and spike selectivity as the measured values. The firing probability computed using the conductance pair with the average inhibition and the preferred excitation was 0.9, whereas, the firing probability computed from the conductance pair with the null excitation was 0 (SI = 1). In other words, whatever contribution inhibition made to synaptic selectivity in this example, that contribution was undetectable in the spike counts.

We next assessed the extent to which inhibition's role in modulating input strength affected spike selectivity by asking how eliminating inhibition affected the difference between the preferred and null spikes. Without inhibition, the preferred excitation evoked an average of 1.76 spikes per stimulus, whereas the null firing count increased to 0.96, reducing the SI to 0.29 (Figure 3E). Note that inhibition enhanced spike selectivity in two ways that are consistent with gain control. First, it kept the null response below threshold, i.e., the null gain = 0. Also, the null firing increased slightly more than the preferred following inhibitory block. More importantly, because the SI is calculated as a ratio, the overall increase in firing reduced the SI. This points out that by limiting the overall firing rates, inhibition can increase the relative difference in spiking, even if it does not alter the absolute spiking difference.

We conclude that in this example, inhibition enhanced spike selectivity by controlling the gain and by limiting firing rates rather than by its contribution to synaptic selectivity. Furthermore, the effects of removing inhibition on spike rate and spike selectivity are consistent with the effects commonly observed when inhibition is blocked *in vivo*. Although, this example is representative of most of our data, there are cells in which inhibition's contribution to synaptic selectivity does affect spike selectivity, as shown in the next section.

### Summary data

A summary of the eight conductance sets from the five cells is shown in Figure 4. On average, the spike counts evoked by current steps computed from the model cells were similar to the measured spike counts (Figure 4A). The responses computed from the FM-evoked conductance waveforms matched the measured FM-evoked firing probability closely (Figure 4B). In 14/16 conductance pairs, the computed and measured firing probabilities were within 10%. In one conductance set, the computed firing probability to the preferred conductance pair was 0.96, lower than the measured probability of 1.4. In another set, the null-computed spiking probability was 0.7 compared to the measured 0.5.

In terms of directional selectivity, the model cells and measured data were also similar (Figure 4C). We show the computed SI plotted against the measured SI for each conductance set. In five out of eight sets, the computed and measured SIs were equal. In three sets, the computed SI was slightly lower than measured. Since blocking inhibition typically reduces the SI, a low computed SI for the control condition is a conservative error. Taken together, the models are a good representation of the individual cells.

To determine the extent to which inhibition enhanced spike selectivity by contributing to synaptic selectivity or gain control, we computed firing probabilities and SIs using either the average of the preferred and null inhibitions or by eliminating inhibition. We first show the effect of eliminating inhibition on firing probability (Figures 4D,E).

Eliminating inhibition increased spike probability in almost every case (Figure 4D), and inhibition kept the null responses below threshold (gain = 0) in four out of eight sets. Furthermore, the increase in firing probability, the difference between the firing probability with and without inhibition, was greater in the null than in the preferred in six out of eight sets (Figure 4E). Thus, inhibition affected firing rates and the gain in most cases.

We next assessed the effect on spike selectivity of inhibition's contribution to synaptic selectivity compared with inhibition's contribution to gain control (Figure 4F). For each conductance set, we show the SI computed with the control excitation combined with either the control inhibition (control), the average inhibition or with no inhibition. In five out of eight cases, using the average inhibition did not alter the SI (red horizontal lines, four cases are overlapping), indicating that any contribution that inhibition made to synaptic selectivity was undetectable in the spike counts. In these five cases, the SI was not reduced until inhibition was eliminated. In the remaining three cases, eliminating the inhibitory differences reduced the SI. However, eliminating inhibition entirely reduced the SI even more, indicating that inhibition contributed to spike selectivity by enhancing synaptic selectivity and as a gain control in these three cells. Thus, inhibition sometimes enhanced spike selectivity by enhancing synaptic selectivity (three out of eight), but always enhanced spike selectivity as a gain control (eight out of eight).

## Discussion

### Summary

Using modeling and results from *in vivo* whole-cell recordings, we investigated the role of inhibition in shaping neuronal response selectivity in the IC. The first result is that changes in spike selectivity following inhibitory block do not necessarily distinguish amongst potential underlying mechanism(s) of spike selectivity. This was shown in two distinct cell types, sustained cells and onset cells, which represent the extremes of the cell types found in the IC in terms of input/output functions. Using empirically derived synaptic inputs and modeling, we found that when inhibitory block reduced or eliminated spike selectivity, inhibition sometimes enhanced spike selectivity by contributing to synaptic selectivity, but always by modulating the firing rates and gain. These inhibitory mechanisms are not mutually exclusive. We first discuss the simplicity of the model cells, then briefly review the terms gain and gain control in the context of enhancing spike selectivity.

### Simple model

Although, the model cells in this study are very simple, they captured the essential empirically measured features, and we do not believe that a more complex or physiologically accurate model would change the central conclusions. For example, as noted in the methods, the low-voltage-activated potassium conductance in the onset cell model was not physiological. In order to fire a few spikes rather than a single spike at the start of prolonged depolarization, we mathematically delayed the activation of a KLT current. A more physiologically accurate approach would have been to include additional currents, such as, a Ih or a *t*-type calcium current (Sivaramakrishnan and Oliver, 2001; Koch and Grothe, 2003). However, modeling studies have shown that many different combinations of intrinsic properties can achieve the same I/O curve (Prinz et al., 2003), and suggest that similar I/O curves would be functionally similar independent of the underlying intrinsic properties. Also, we would expect that a multi-compartment model with the appropriate complement of intrinsic properties might show a greater role for synaptic selectivity in creating differences between preferred and null PSPs (PSP selectivity). For example, Ih in cortical dendrites strongly affects the temporal summation of inputs, and is not homogenously expressed (Magee, 1998). Due to space clamp issues, such dendritic processing might be undetectable in somatic recordings, even with channel blockers (Williams and Mitchell, 2008). Although, we have argued that the small size and high input resistance of most IC cells likely reduces the space clamp problem in IC cells compared to cortical neurons (Gittelman and Li, 2011), processing in the dendrites could be undetected or underestimated. However, we suggest that inhibition would function similarly in the dendrites with the exception that the output might be PSPs instead of spikes, i.e., there would be no spike threshold. Nevertheless, inhibition would keep PSP size small and away from saturation. Whatever processing occurs in the dendrites would then be passed as input to the next compartment (e.g., the soma).

### Gain and gain control

Before illustrating how inhibition modulates gain and how this affects spike selectivity, we note that gain and gain control have been described in a number of different ways in the literature. In order to clarify these terms, we consider a neuronal I/O curve with current as the input and spikes as the output (Figure 5). Neuronal I/O curves are typically sigmoidal in that in response to small (subthreshold) inputs the output is zero. As the current strength increases, the output increases up to a point, and then eventually saturates. It is often the case that continuing to increase the current strength will eventually decrease the output (not shown).

Gain is the ratio of output/input (y/x), and therefore, the gain changes with input strength. At the far left of the curve, when the output is zero, the gain is zero. As the current strength increases, the I/O curve enters a rising phase during which output increases approximately in proportion to input. During this rising phase, the gain increases rapidly at first, but then more slowly as saturation is approached. During the saturation phase (far right), gain decreases as input strength increases, but does not return to zero.

One definition of gain control, termed additive/subtractive, is a horizontal shift of the I/O curve (Ingham and McAlpine, 2005). With only excitatory current as the input (X-axis), inhibition shifts the I/O curve rightward (see Figure 9, Ingham and McAlpine, 2005). However, the input to a cell is not just excitation, but also includes inhibition. Therefore, the input is better described as total current rather than just excitatory current. With total current as the input, inhibition does not change the I/O curve, rather it changes the input strength (Holt and Koch, 1997; Chance et al., 2002). In this mathematically equivalent view, inhibition works as an additive/subtractive gain control by shifting the input strength leftward along the X-axis (see section “How Modulating Gain Affects Spike Selectivity”).

Another definition of gain that is often used in neurobiology is the slope of the I/O curve (dy/dx), i.e., the proportional change of output with respect to input (Isaacson and Scanziani, 2011). To distinguish between this definition and the preceding definition (y/x), we refer to dy/dx as the proportional gain. The proportional gain also changes with input strength, but in a different way. The proportional gain is zero at the far left of the curve when the output is zero. It increases at the start of the rising phase, peaks at the midpoint of the rising phase, and then decreases as saturation is approached, rapidly returning zero.

Also in neurobiology, it is often the case that only the rising phase of the I/O curve is considered in discussions of proportional gain (Isaacson and Scanziani, 2011). As the rising phase can be fit reasonably well with a line, the proportional gain is considered constant. Gain control is then defined as a change in the slope of that rising phase (Chance et al., 2002; Ingham and McAlpine, 2005). Termed multiplicative or divisive gain control, this is a useful definition when considering, for example, how attention changes spike selectivity with respect to an environmental stimulus rather than with respect to current (McAdams and Maunsell, 1999; Treue and Martínez Trujillo, 1999). However, without considering the entire I/O curve it is not possible to describe the multiple ways in which inhibition modulates the gain, the proportional gain, and spike selectivity. When the entire curve is considered, and input is defined as current, inhibition modulates the gain and the proportional gain by shifting the net input current leftward with no concomitant change to the slope of the I/O curve (Holt and Koch, 1997; Chance et al., 2002). As we describe next, these changes in gain can account for changes in spike selectivity. Importantly, they can also account for the multiplicative/divisive gain control observed when the input (X-axis) is an environmental stimulus rather than current (Murphy and Miller, 2003).

### How modulating gain affects spike selectivity

How inhibition alters gain and spike selectivity depends on where the inputs fall along the I/O curve, and where they move when inhibition is removed. We illustrate how inhibition can shape spike selectivity without contributing to synaptic selectivity using a schematic (Figure 5). In each scenario, we assume that the difference between the preferred and null input currents is due to differences between the preferred and null excitations, i.e., synaptic selectivity relies entirely on excitation to generate input selectivity. The preferred and null inhibitions are presumed identical. Although, not strictly true, we conservatively assume that identical inhibitions would shift the inputs leftward equally along the X-axis (and thus blocking inhibition would shift the inputs equally rightward). At typical resting potentials, identical inhibitions would actually shift the null slightly more along the X-axis than it would shift the preferred. In most situations, this would mean that inhibition enhances spike selectivity more than if the lateral shifts were exactly equal.

In the first scenario, inhibition enhances spike selectivity by keeping the inputs below saturation (Figure 5A). Under control conditions, the inputs fall within the rising phase, with high proportional gain. The preferred input (tall, thick dashed line) is larger than the null (short, thin dashed line), and therefore, evokes more spikes compared to the null. Once inhibition is removed, both inputs shift rightward equally, such that they both evoke the maximum number of spikes, i.e., the output saturates (red lines), and spike selectivity is eliminated. In other words, inhibition keeps the inputs within the rising portion of the I/O curve, where the gains are high, and the gain of the preferred is larger than the null gain. The steep slope (high proportional gain) results in even small differences between the preferred and null inputs translating into differences in the spiking. When inhibition is removed in this example, the inputs shift rightward to a region of the I/O curve where the preferred gain is less than the null gain, and the proportional gain goes to zero. Consequently, no matter how large the difference between the preferred and null inputs, they will evoke the same number of spikes. This, and each subsequent example, shows an example of inhibition enhancing spike selectivity by functioning as a subtractive gain control. By reducing input strength, inhibition changed the gains of the preferred and null inputs, and consequently increased the difference in output spikes.

There is a corollary to this example in which the inputs shift rightward to a region of gentler slope, but do not reach saturation (Figure 5B). It should be clear that without reaching saturation, the preferred proportional gain decreases more than the null proportional gain. Consequently, the same difference between the preferred and null inputs would generate a smaller difference in spiking, i.e., spike selectivity would decrease even without reaching saturation.

Consistent with the above ideas, studies in the IC show that inhibition prevents spike rate from approaching saturation (Sivaramakrishnan et al., 2004; Ingham and McAlpine, 2005). This is also true in the cortex, where inhibition prevents epileptiform activity (Dudek and Sutula, 2007; Katzner et al., 2011), although, the phenomenon is not typically described as gain control.

Additionally, inhibition keeps spike rates low, which also enhances spike selectivity (Figure 5C). Under control conditions the preferred input is larger than the null, but both fall within the rising phase of the I/O function. When inhibition is removed, both shift equally to the right, but stay within the rising phase. In this example the proportional gain does not change (much). However, the preferred and null gains do change. With inhibition, the preferred gain is substantially larger compared to the null gain. When inhibition is removed, both the preferred and the null gain increase, but the null gain increases more compared to the preferred. In other words, the difference between the preferred and null gain is reduced. Because the SI is a ratio, it decreases when the preferred and null spikes increase equally.

Finally, inhibition enhances spike selectivity by keeping the null input below spike threshold (Figure 5D, Rose and Blakemore, 1974; Wehr and Zador, 2003; Gittelman et al., 2009; Katzner et al., 2011). The null evokes no spikes under control conditions, and therefore, the null gain is zero. When inhibition is removed, both the preferred and null inputs shift equally to the right, and the null exceeds threshold (null gain > 0). Although this may increase the absolute difference between the preferred and null spikes, it nevertheless reduces spike selectivity as measured by the SI [SI = (P−N)/(P+N), where P is the preferred spike count and N is the null spike count]. Under control conditions the null spike count N = 0, and so the SI = 1. After inhibition is blocked, N > 0, so SI < 1.

What this suggests is that when quantified by a SI, nearly any change in firing rates will change the SI, and in most situations, increasing firing rates will reduce it. Blocking inhibition almost always increases firing rates in the IC, and therefore, almost always reduces the SI. What is important here is that this is true independent of whether or not inhibition contributes to synaptic selectivity.

### Synaptic selectivity vs. gain control

Evidence suggests that inhibition contributes to synaptic selectivity in the IC, but this contribution only enhances spike selectivity in some cells or perhaps to particular stimuli. A clear example from extracellular recordings is for non-monotonic rate level functions, in which firing rates increase initially as sound intensity increases, but the rates decrease as sound intensity continues to increase. In addition to increasing overall firing rates, blocking inhibition often converts this profile to monotonic, in which firing rates increase continuously or saturate as the sound level increases, but do not decrease (Park and Pollak, 1993; Fuzessery and Hall, 1996; Sivaramakrishnan et al., 2004). The simplest interpretation of these results is that high intensities recruit additional inhibition that suppresses the spiking response under control conditions. Consider the medium intensity sound as the preferred signal, and the high intensity sound as the null. In this case, inhibition enhances spike selectivity by contributing to the synaptic selectivity: the null stimulus evoked more inhibition compared to the preferred stimulus. However, inhibition also changes the input strength, and so likely enhances spike selectivity by subtractive gain control (see section “Gain and Gain Control”).

The most direct evidence for inhibition contributing to synaptic selectivity comes from the few *in vivo* whole cell recordings in the IC in which the excitatory and inhibitory inputs are at least partially isolated. Inhibition contributes to synaptic selectivity for FM sweeps, at least in some cells (Gittelman et al., 2009; Gittelman and Li, 2011; Kuo and Wu, 2012). In one recent study, Kasai et al. (2012) found the clearest evidence for inhibition creating selective responses to the offset of tones, possibly in the absence of excitation. These cells fired selectively to the offset of a preferred tone duration, i.e., the preferred duration evoked a robust response, whereas a null duration evoked fewer or no spikes. In two of the 11 offset neurons, the selective firing was due to a (presumably) inhibition-induced hyperpolarization, combined with intrinsic properties that facilitate anode-break excitation. Although, the separation of excitation and inhibition was not perfect, no evidence of excitation was found, suggesting that it was small if present. In other words, inhibition was the major—and possibly only—contributor to synaptic selectivity in these two cells.

Interestingly, it has now been demonstrated in the mammalian auditory system that excitation is not necessary for selective spiking, at least in the superior paraolivary nucleus (SPN). Kopp-Scheinpflug et al. (2011) showed *in vitro* that offset responses can be evoked by inhibitory transmission that hyperpolarizes the cell along with Ih and low-threshold *t*-type calcium currents that facilitate rebound excitation. Although it is not clear how common this mechanism is *in vivo*, the study nicely demonstrates that under certain conditions no excitation is required to evoke spikes. Biophysically, the onset cells in the IC are similar to those in the SPN (Koch and Grothe, 2003; Xie et al., 2008). Combined with the observations of Kasai et al. (2012) in which no evidence of excitatory input was found in duration-tuned cells of the IC, there is a possibility that the same mechanism operates in the IC. This may be the most extreme case possible, in which inhibition is the only synaptic input, and therefore, is the exclusive contributor to synaptic selectivity.

Having noted that inhibition enhances spike selectivity by contributing to synaptic selectivity in some situations, several studies in which spike selectivity was measured in the IC before and after inhibitory block using extracellular electrodes have proposed that inhibition increases selectivity by gain control (Ingham and McAlpine, 2005; Pérez-González et al., 2012). Following inhibitory block, tuning for interaural phase differences (IPD) is reduced and the overall firing rates increase, without a change in the best IPD (Ingham and McAlpine, 2005). The authors suggest one way to explain these changes is by the mechanisms in Figure 5, in which inhibition operates as a subtractive gain control. Similarly, the effects of blocking inhibition on stimulus specific adaptation (SSA) are consistent with inhibition functioning as a subtractive gain control (Pérez-González et al., 2012). Firing rates to the high-probability standard tone (null-like) and to the low-probability deviant tone (preferred-like) increased during inhibitory block, which reduced the SSA selectivity index (also a ratio), consistent with the model in Figure 5C. There were also cases in which inhibition eliminated the response to the standard tone, i.e., kept it below threshold, as in Figure 5D.

Evidence from whole-cell recordings in the IC suggests it is very rare for a sound to evoke excitation without evoking inhibition. As noted above, sometimes inhibition is evoked in the absence of excitation (Xie et al., 2007; Kuo and Wu, 2012), and this occasionally evokes spikes (Kasai et al., 2012). However, in general, sounds that drive spikes evoke both excitation and inhibition, consistent with a role for inhibition in enhancing spike selectivity by modulating firing rates and gain. What is most striking is that even when inhibition reduces the difference between the preferred and null PSPs, it can still enhance spike selectivity by the mechanisms shown in Figure 5 (Gittelman et al., 2009; Gittelman and Li, 2011). As noted at the end of section “How Modulating Gain Affects Spike Selectivity,” when inhibition is present with excitation, it nearly always enhances spike selectivity by reducing firing rates and/or by subtractive gain modulation.

Finally, we note that inhibition performs these same functions in the cortex (see Isaacson and Scanziani, 2011, for an excellent review). Whole-cell recordings *in vivo* have found evidence that inhibition enhances synaptic selectivity in the cortex in some cells and for some stimuli (Anderson et al., 2001; Zhang et al., 2003; Tan et al., 2004; Wu et al., 2006; Ye et al., 2010). But the majority of studies suggest that in most cells, excitation and inhibition are approximately co-tuned and approximately balanced, in that excitation and inhibition have very similar receptive fields, and that the ratio of excitation to inhibition is roughly constant (Anderson et al., 2000; Wehr and Zador, 2003; Tan et al., 2004; Priebe and Ferster, 2005; Okun and Lampl, 2008). These characteristics are consistent with inhibition reducing synaptic selectivity, but functioning as a gain control. Furthermore, inhibition enhances spike selectivity in the cortex by preventing hyperexcitability (Dudek and Sutula, 2007; Katzner et al., 2011), and often by keeping null inputs below threshold (Rose and Blakemore, 1974; Wehr and Zador, 2003). Although most cortical studies limit the definition of gain control to “multiplicative/divisive” gain control as described in section “Gain and Gain Control,” and therefore do not refer to these mechanisms as gain control (Isaacson and Scanziani, 2011), functionally these are the same roles served by inhibition in the IC. Although data in the IC are limited, they suggest that inhibition enhancing spike selectivity by controlling the gain may be a general rule rather than an exception.

### Conflict of interest statement

The authors declare that the research was conducted in the absence of any commercial or financial relationships that could be construed as a potential conflict of interest.
